# Glucocorticoid maturation of mitochondrial respiratory capacity in skeletal muscle before birth

**DOI:** 10.1530/JOE-21-0171

**Published:** 2021-07-27

**Authors:** K L Davies, E J Camm, D J Smith, O R Vaughan, A J Forhead, A J Murray, A L Fowden

**Affiliations:** 1 Department of Physiology, Development and Neuroscience, University of Cambridge, Cambridge, UK; 2 The Ritchie Centre, Hudson Institute of Medical Research, Clayton, Australia; 3 Institute for Women’s Health, University College London, London, UK; 4 Department of Biological and Medical Sciences, Oxford Brookes University, Oxford, UK

**Keywords:** mitochondria, cortisol, maturation, fetus

## Abstract

In adults, glucocorticoids act to match the supply and demand for energy during physiological challenges, partly through actions on tissue mitochondrial oxidative phosphorylation (OXPHOS) capacity. However, little is known about the role of the natural prepartum rise in fetal glucocorticoid concentrations in preparing tissues for the increased postnatal energy demands. This study examined the effect of manipulating cortisol concentrations in fetal sheep during late gestation on mitochondrial OXPHOS capacity of two skeletal muscles with different postnatal locomotive functions. Mitochondrial content, biogenesis markers, respiratory rates and expression of proteins and genes involved in the electron transfer system (ETS) and OXPHOS efficiency were measured in the *biceps femoris* (BF) and *superficial digital flexor* (SDF) of fetuses either infused with cortisol before the prepartum rise or adrenalectomised to prevent this increment. Cortisol infusion increased mitochondrial content, biogenesis markers, substrate-specific respiration rates and abundance of ETS complex I and adenine nucleotide translocator (ANT1) in a muscle-specific manner that was more pronounced in the SDF than BF. Adrenalectomy reduced mitochondrial content and expression of *PGC1α* and *ANT1* in both muscles, and ETS complex IV abundance in the SDF near term. Uncoupling protein gene expression was unaffected by cortisol manipulations in both muscles. Gene expression of the myosin heavy chain isoform, *MHCIIx,* was increased by cortisol infusion and reduced by adrenalectomy in the BF alone. These findings show that cortisol has a muscle-specific role in prepartum maturation of mitochondrial OXPHOS capacity with important implications for the health of neonates born pre-term or after intrauterine glucocorticoid overexposure.

## Introduction

In adults, glucocorticoids are stress hormones with metabolic actions on a wide range of tissues that maintain functions critical to survival in adverse environmental conditions and during normal physiological challenges to homeostasis-like exercise and pregnancy (Picard *et al.* 2018, Bartho *et al.* 2020, Casuro & Huertus 2020). Many of these functions require energy in the form of ATP, which is produced mainly by oxidative phosphorylation (OXPHOS) in the mitochondria (Nunnari & Suomalainen 2012, Rodriguez-Cano *et al.* 2020). Mitochondria, therefore, have a key role in the response to both internal and external environmental cues and are known to be regulated by glucocorticoids in adulthood (Lee *et al.* 2013, Lapp *et al.* 2019).

Mitochondria are dynamic organelles that respond to changes in energy demand by biogenesis, fusion/fission and by alterations in the abundance of the electron transfer system (ETS) complexes and other proteins regulating ATP production (Goffat & Wiesner 2003, Liang & Ward 2006, Chandhol *et al.* 2018). Utilising a range of metabolic substrates, ATP is produced by ATP synthase using the proton gradient across the inner mitochondrial membrane generated by redox reactions at ETS complexes with oxygen as the final electron acceptor. The efficiency of mitochondrial OXPHOS also depends on uncoupling proteins (UCPs) that dissipate the proton gradient when activated, and on transporters that shuttle adenine nucleotides across the mitochondrial membranes (Kimura & Rasmussen 1977, Nunnari & Suomalainen 2012). Glucocorticoids have been shown to influence many of these regulatory processes in mitochondria of several adult tissues, including skeletal muscle (Djouadi *et al.* 1994, Rachamim *et al.* 1995, Weber *et al.* 2002, Du *et al.* 2009).

Glucocorticoids can also act as stress signals in the fetus but, during normal conditions in late gestation, their primary role is as a signal of impending delivery (Reynolds 2013, Fowden & Forhead 2015). In most mammals studied to date, fetal glucocorticoid concentrations rise naturally towards term and switch fetal tissues from growth to differentiation in preparation for birth (Fowden *et al.* 1998). This prepartum glucocorticoid surge also activates many processes that have little or no function *in utero* but which are essential for neonatal survival such as breathing, thermogenesis, glucogenesis and locomotion (Fowden *et al.* 2016). All these new functions require extra energy but relatively little is known about the effects of glucocorticoids on mitochondrial function in fetal tissues during late gestation, particularly in species that are mobile from birth.

In several species, mitochondrial function is known to rise between fetal and neonatal life in several different tissues (Prieur *et al.* 1998, Lehman *et al.* 2000, Nakai *et al.* 2002, Minai *et al.* 2008, Rog-Zielinska *et al.* 2015, Davies *et al* 2020). Administration of potent synthetic glucocorticoids during rodent pregnancy has also been shown to affect the abundance of mitochondrial proteins in fetal tissues near term (Nakai *et al.* 2002, Prieur *et al.* 1998, Rog-Zielinska *et al.* 2015). In addition, a recent study in fetal sheep has demonstrated that the natural prepartum cortisol surge closely parallels the increase in mitochondrial OXPHOS capacity of skeletal muscle towards term (Davies *et al.* 2020). However, whether these changes are the direct consequence of the fetal cortisol increment remains unknown. This study, therefore, examined the hypothesis that cortisol causes maturation of mitochondrial OXPHOS capacity in skeletal muscle towards term.

## Methods

### Animals

A total of 24 time-mated pregnant ewes and 6 newborn twin lambs were used in this study. Of the pregnant ewes, 12 carried single fetuses while the remainder were twin-bearing. Pregnant ewes were group-housed before surgery and singly housed within sight and sound of other sheep after surgery. They had free access to hay and water at all times except for 12–18 h before surgery when food was withheld. All animal procedures were carried out under the UK Animals (Scientific Procedures) Act 1986 Amendment Regulations 2012 following ethical review by the University of Cambridge Animal Welfare and Ethical Review Body.

### Surgical procedures

Between 114 and 119 days of gestational age ( dGA), surgery was carried out on 6 twin-bearing and 12 single-bearing ewes under isofluorane anaesthesia (1.5–2% in 5:1 O_2_:N_2_O mixture) with positive pressure ventilation. In twin-bearing ewes, one fetus was adrenalectomised (AX) and its twin was sham-operated as a control (Barnes *et al.* 1978). In the single-bearing ewes, catheters were inserted into the maternal dorsal aorta and the fetal dorsal aorta and caudal vena cava, via the femoral vessels, and exteriorised through the maternal flank (Fowden & Silver 1995). The ewes were monitored throughout surgery using a capnograph and pulse oximeter. At surgery, the ewes were given antibiotics (oxytetracycline, 20 mg/kg i.m., Allamycin, Norbrook Laboratories, Newry, UK and penicillin, Depocillin, Intervet international, Milton Keynes, UK, 15 mg/kg i.m. to mother and intra-amniotically or i.v. to fetus) and analgesia (1 mg/kg carprofen, s.c. Rimadyl, Zoetis, London UK). Penicillin treatment to the ewe continued for 2 days post-operatively.

### Experimental procedures

All catheterised animals were sampled daily to maintain catheter patency and to collect blood samples to measure blood gases and concentrations of metabolites and hormones. Following post-operative recovery for at least 5 days, the catheterised fetuses were assigned randomly to receive a 5-day i.v. infusion of either saline (0.9% NaCl, 3 mL/day, *n* = 6, control, 3 male M: 3 female F) or cortisol (2–3 mg/kg/day Solu-Cortef; Pharmacia, *n* = 6, 4M:2F). At the end of infusion (128–131 dGA), the ewes and fetuses were killed by administration of a lethal dose of anaesthetic (200 mg/kg sodium pentobarbitone, iv, Pentoject, Animalcare Ltd, York, UK) and tissues collected from the fetus. Similarly, at 141–145 dGA, the ewes with AX (4M:2F) and sham-operated fetuses (2M:4F) were euthanised with an overdose of anaesthetia as above and the fetuses were delivered in random order. A blood sample was taken from the umbilical artery of each fetus before administration of a lethal dose of sodium pentobarbitone (200 mg/kg) and tissue collection. At delivery, the two female AX fetuses had small adrenal remnants (80 mg and 180 mg) so neither was used for any subsequent analyses.

Umbilical arterial blood and skeletal muscle were also collected from twin fetuses of six unoperated ewes at 102–105 dGA as described above. Tissue from only one fetus of each pair (2M:4F) was randomly selected for further study. In addition at 1–2 days of postnatal age, one lamb from six unoperated pairs of twins (3M:3F) was euthanised for tissue collection using sodium pentobarbitone (200 mg/kg) after collection of a blood sample from the jugular vein. All blood samples were collected into heparin-coated tubes and, after centrifugation, the plasma was stored at −20°C for future hormone analysis. Immediately following euthanasia, the fetal and newborn lambs were weighed and measured.

Two hindlimb skeletal muscles with different postnatal functions in locomotion, the *biceps femoris* (BF) and *superficial digital flexor* (SDF), were immediately collected and weighed. The BF is a large powerful, multifunctional muscle producing mechanical power by shortening while the SDF is a small flexor muscle generating force predominately by isometric contraction (Fourie 1962, Biewener 1998). The BF controls locomotive gait through extension and abduction of the hindlimb whereas the SDF controls foot placement important for allowing the neonate to stand (Fourie 1962, Walker & Luff 1995). Both muscles are of mixed fibre type with a combination of slow-twitch type I and fast-twitch type II fibres (Davies 2018). In late gestation, the SDF has proportionally more type I fibres than the BF, although both muscles still contain undifferentiated fibres at birth (Davies 2018, Davies *et al.* 2020).

Samples of these muscles were snap-frozen in liquid nitrogen before being stored at −80°C until required. Additionally, in the fetuses at 129 and 144 dGA, a small sample (≈100–200 mg) from the centre of each muscle was collected into ice-cold biopsy preservation solution (BIOPS; pH 7.1 solution containing 2.77 mM CaK_2_EGTA, 7.23 mM K_2_EGTA, 20 mM imidazole, 20 mM taurine, 50 mM MES, 0.5 mM dithiothreitol, 6.56 mM MgCl_2_.H_2_O, 5.77 mM Na_2_ATP and 15 mM phosphocreatine; Pesta & Gnaiger 2012) before dissection for respirometry.

### Respirometry

Respirometry measurements were made on the skeletal muscle samples from the AX, sham-operated, cortisol- and saline-infused groups of fetuses using the protocol described previously for this tissue (Kuznetsov *et al.* 2008, Pesta & Gnaiger 2012). Briefly, 2–3 mg pieces of tissue were dissected in BIOPS, bundles of 6–8 fibres were teased apart before incubating with saponin for 20 min to permeabilise the plasma membrane (100 µg saponin/mL BIOPS). Samples were transferred into an isotonic respiration medium maintained at 37°C (MiR05; pH7.1 solution containing 20 mM HEPES, 0.5 mM EGTA, 3 mM MgCl_2_.6H_2_O, 10 mM KH_2_PO_4_, 20 mM taurine, 110 mM sucrose, 60 mM K-lactobionate and 1g/l BSA; Pesta & Gnaiger 2012, Gnaiger *et al.* 2000) in order to measure oxygen (O_2_) consumption using Clark-type oxygen electrodes (Strathkelvin Instruments, Glasgow, UK). Substrates were added into the chambers at saturating concentrations according to three protocols as previously described (Davies *et al.* 2020). Malate (2 mM), glutamate (10 mM), ADP (10 mM) and succinate (10 mM) were added in sequence to give a measure of maximal ADP-coupled oxygen consumption when electron entry to both complexes I and II of the ETS is saturated. The second protocol involved the addition of malate (2 mM), pyruvate (5 mM) and ADP (10 mM) was used to obtain a measure of oxidative capacity for pyruvate (Py), a derivative of glucose. Thirdly, malate (2 mM), palmitoyl-carnitine (PC, 40 µM) and ADP (10 mM) were added to provide a measure of fatty acid oxidation capacity. In all protocols, leak state was measured in the presence of substrates before the addition of ADP, and the experiment concluded with the addition of cytochrome c (10 µM) to check outer mitochondrial membrane integrity. Results were excluded if there was a ≥15% increase in O_2_ consumption following cytochrome c addition. Additionally, data were excluded if the rate of O_2_ uptake over the baseline period before substrates were added, exceeded 0.001 µmol O_2_/min as this suggests insufficient plasma membrane permeabilisation (Kuznetsov *et al.* 2008). Following respirometry, muscle fibres were extracted from chambers and dried for 48 h before being weighed, and results are presented as O_2_ consumption/mg dry weight.

### Biochemical analyses

#### Hormone assays

Plasma cortisol concentrations were measured using a human ELISA (RE52061, Tecan, Männedorf, Switzerland), previously validated for sheep plasma (Vaughan *et al.* 2016). Intra- and inter-assay coefficients of variation for the cortisol assay were 3 and 5%, respectively, and the limit of detection was 5.2 ng/mL. Because cortisol increases fetal T_3_ concentrations towards term and thyroid hormones are known to affect O_2_ consumption by fetal tissues (Fowden & Silver 1995, Forhead *et al.* 2006, Davies *et al.* 2020, 2021), total plasma T_3_ and T_4_ were also measured using radioimmunoassays (Kit numbers, 06B254215 and 06B 254011, respectively, MP Biomedical, Eschwege, Germany), previously validated for sheep plasma (Fowden & Silver 1995). Intra- and inter-assay variations were less than 2 and 8% for T_3_ and 3 and 5% for T_4_. The limit of detection was 0.14 ng/mL for T_3_ and 11.3 ng/mL for T_4_.

#### Biochemical composition

Water content was calculated as a percentage by weighing, freeze-drying overnight and then re-weighing samples of frozen muscles. Following extraction from homogenised frozen tissue, protein content was measured using a bicinchoninic acid assay and expressed as mg protein per gram tissue (wet weight) or as mg protein per mg dry weight calculated using the percentage water content of the muscle.

#### Citrate synthase activity

Activity of citrate synthase (CS), an enzyme of the tricarboxylic acid cycle, is a putative marker of muscle mitochondrial content (Larsen *et al.* 2012) and was measured spectrophotometrically in the skeletal muscles. Ten to thirty micrograms of homogenised protein was added to the assay buffer (pH 8) containing 0.1 mM 5,5’-Dithio-bis(2-nitrobenzoic acid), DTNB, 1 mM oxaloacetate and 0.3 mM acetyl-CoA. CS activity was determined as the maximal rate of absorbance change at 412 nm over 3 min (a measure of the rate of 5-thio-2-nitrobenzoic acid production). CS activity is expressed as per mg protein.

#### Western blotting

Frozen muscle samples (55 mg ± 10%) were homogenised, total protein extracted and diluted to 2.5 µg/µL in 8% SDS solution. Protein was electrophoresed on a 12% polyacrylamide gel, transferred to a nitrocellulose membrane and stained with Ponceau-S to allow for normalisation of protein loading. Membranes were incubated either with primary antibodies to ETS complexes I-IV and ATP synthase (OXPHOS antibody cocktail; 458099; Life Technologies; 1:1000), followed by an HRP-linked anti-mouse secondary antibody (NIF82; GE Healthcare; 1:5000) or to ANT1 (Abcam, ab1002032, 1:1000), followed by HRP-linked donkey anti-rabbit IgG (GE healthcare; NA934V, 1:5000). ECL was used to visualise protein bands and quantified using ImageJ (http://rsb.info.nih.gov/ij/).

#### qRT-PCR

Frozen skeletal muscle samples were powdered and RNA extracted using TRIzol (Thermo Fisher) and chloroform, and the aqueous phase used in the RNeasy Plus kit (Qiagen). RNA concentration was measured using a Nanodrop ND-1000 spectrophotometer, diluted to 50 ng/µL and used for cDNA synthesis (High Capacity cDNA RT Kit; Applied Biosystems). qRT-PCR was performed using a MESA BLUE Mastermix (Eurogentec, Liège, Belgium) following the manufacturer's recommended protocol (5 min at 95°C followed by 40 amplification cycles of 15 s at 95°C and 1 min at 60°C). The genes assayed, their encoded protein and function together with the primer sequences used are given in Table 1. Results were analysed using 2^−ΔΔCt^ method (Schmittgen & Livak 2018) and expressed relative to the geometric mean of S15 and 18S housekeeper genes and set relative to the average of the relevant control group. All samples were run in triplicate and housekeeper gene expression did not differ significantly between groups.

**Table 1:** Forward and reverse primer sequences used for SYBR qRT-PCR.

Target gene, encoded protein and function	Primer sequences	Reference
*Ribosomal protein S15 (RPS15)*	F: ATCATTCTGCCCGAGATGGTG	Yates *et al.* 2016
	R: TGCTTCACGGGCTTGTAGGTG	
*18S rRNA*	F: GTAACCCGTTGAACCCCATT	Byrne *et al.* 2010
	R: CCATCCAATCGGTAGTAGCG	
*Peroxisome proliferator-activated receptor gamma coactivator 1 alpha (PPARGC1A*)		
PGC1α protein	F: GAGATGTGACCACCGAGAATGAG	Myers *et al.* 2008
Regulator of Mitochondrial biogenesis	R: GCTGTTGACAAATGCTCTTCGC	
	R: CACCGCCGAATAATTCACTT	
*Mitofusin 2 (MFN2)*		
MFN2 protein	F: CATCAGCTATACTGGCTCCAACT	Davies *et al.* 2020
Regulator of mitochondrial membrane fusion	R: AATGAGCAAAAGTCCCAGACA	
*Dyamin-related protein1 (DRP1)*		
DRP1 protein	F: ATGCCAGCAAGTCCACAGAA	Reddy *et al.* 2016
Regulator of mitochondrial membrane fission	R: TGTTCTCGGGCAGACAGTTT	
*Uncoupling protein 2 (UCP2)*		
UCP2 protein	F: AAGGCCCACCTAATGACAGA	Davies *et al.* 2020
Mitochondrial uncoupling	R: CCCAGGGCAGAGTTCATGT	
*Uncoupling protein 3 (UCP3)*		
UCP3 protein	F: GAAAGGAATTCTGCCCAACA	Kelly *et al.* 2011
Mitochondrial uncoupling	R: TCCAAAGGCAGAGACGAAGT	
*SLC25A4*		
Adenine nucleotide translocase 1 (ANT1) protein	F: TGGTGTCCTACCCCTTTGAC	Kelly *et al.* 2011
Transport of ADP and ATP across mitochondrial membranes. Mild mitochondrial uncoupling	R: CAGGCGCCTTTGAAGAAAGC	
*Myosin heavy chain 7 (MHY7*)		
MHCI protein	F: GAGATGGCCGCGTTTGGGGAG	Yates *et al.* 2016
Muscle contraction	R: GGCTCGTGCAGGAAGGTCAGC	
*MHY2*		
MHCIIa protein	F: ACCGAAGGAGGGGCGACTCTG	Yates *et al.* 2016
Muscle contraction	R: GGCTCGTGCAGGTGGGTCATC	
*MHY1*		
MHCIIx protein	F: AAAGCGACCGTGCAGAGCAGG	Yates *et al.* 2016
Muscle contraction	R: GGCTCGTGCAGGTGGGTCATC	

### Statistical analyses

Data are presented as mean ±s.e.m., and GraphPad Prism Version 6.05 (www.graphpad.com) was used for analyses. A one-way ANOVA was used to assess the developmental changes in CS and plasma cortisol concentration data followed by Tukey’s multiple comparison *post hoc* test. A *t*-test or non-parametric Mann–Whitney test, as appropriate, was used to compare the data between sham-operated and AX and between cortisol- and saline-infused fetuses. Where appropriate, a *t*-test of the significance of a single mean was used to assess the mean difference between the AX and sham-operated twin pairs. Pearson’s correlation coefficient was used to assess correlations between variables and log-transformed hormone concentrations. Partial correlation analysis was applied to determine the relationship between two variables controlling for a third. *P* ≤ 0.05 was considered significant throughout.

## Results

### Hormone concentrations, morphometry and body composition

**Figure 1:**
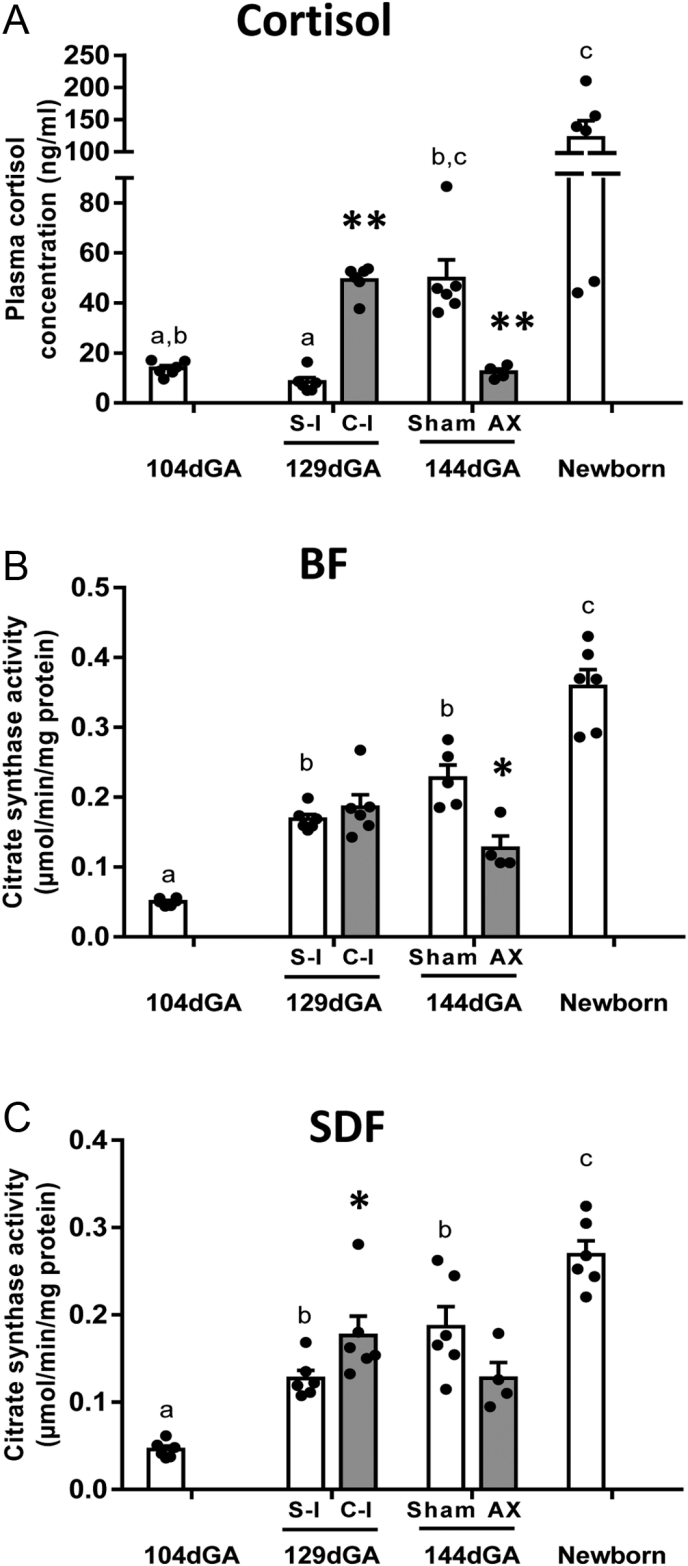
Individual and mean (±s.e.m.) values of (A) fetal cortisol concentration and the activity of citrate synthase (CS) in (B) the *biceps femoris* (BF) and (C) *superficial digital flexor* (SDF) muscles of unoperated newborn lambs (*n* = 6) and fetal sheep delivered either unoperated at 104 days of gestational age ( dGA, *n* = 6), at 129 dGA after infusion with saline (S-I, *n* = 6) or cortisol (C-I, *n* = 6) for 5 days before delivery at 129 dGA or at 144 dGA after adrenalectomy (AX, *n* = 4) or sham operation (Sham, for cortisol n = 6, for CS *n* = 5 BF, *n* = 6 SDF) at 114–119 dGA. Mean (±s.e.m.) values for control animals (104 dGA, S-I, sham-operated and newborn animals) are shown with white columns while those for animals with cortisol concentrations that were manipulated experimentally (C-I and AX) are shown with grey columns. Control columns with different letters as superscripts are significantly different from each other (one-way ANOVA, *P* < 0.05). An asterisk indicates a significant difference from the respective control group (*P < 0.05, **P < 0.01, *t*-test or Mann–Whitney Rank sum test).

In line with previous findings (Barnes *et al.* 1978, Fowden *et al.* 1998), cortisol concentrations increased in control animals towards term and on into the immediate neonatal period (Fig. 1A). Relative to saline-infused fetuses at 129 dGA, cortisol infusion significantly increased the cortisol concentration to values similar to those seen in the older sham-operated controls at 144 dGA (Fig. 1A). In contrast, AX prevented the normal prepartum rise in fetal cortisol concentrations; the mean value in AX fetuses was significantly lower than the concentrations in sham-operated controls at 144 dGA and similar to control values at the earlier gestational ages (Fig. 1A). Fetal plasma T_3_ concentrations were significantly higher in cortisol – than saline-infused fetuses but were not significantly affected by AX, although values had a tendency to be lower in the AX than sham-operated fetuses (*P* = 0.057, Table 2). There were no changes in fetal plasma T_4_ concentrations with cortisol infusion or AX (Table 2).

**Table 2:** Hormone concentrations, morphometry and biochemical composition. Mean (±s.e.m.) values of T3 and T4 concentrations, morphometric measurements and muscle biochemical composition of the biceps femoris (BF) and superficial digital flexor (SDF) muscles of sheep fetuses delivered either at 129 days of gestational age (dGA) after a 5-day infusion of cortisol (*n* = 6) or saline (*n* = 6) or at 144 dGA after adrenalectomy (*n* = 4, AX) or sham operation (Sham, *n* = 6) at 114–119 dGA.

	129 dGA		144 dGA	
	Saline-infused	Cortisol-infused	Sham	AX
Thyroid hormone concentrations				
Plasma T_3_ (ng/mL)	0.43 ± 0.03	0.70 ± 0.14^a^	0.51 ± 0.09	0.23 ± 0.03^b^
Plasma T_4_ (ng/mL)	122.0 ± 9.5	131.6 ± 4.0	98.0 ± 19.0	101.3 ± 13.1
Morphometry				
Body weight (kg)	3.1 ± 0.1	3.0 ± 0.1	3.63 ± 0.27	3.91 ± 0.29
Crown-rump length (cm)	43.3 ± 0.6	43.8 ± 0.5	48.1 ± 0.9	48.8 ± 1.1
Abdominal girth (cm)	33.4 ± 0.6	33.2 ± 1.0	35.3 ± 1.2	37.4 ± 2.5
BF weight (g)	13.07 ± 0.61	12.30 ± 0.87	15.2 ± 1.5	15.2 ± 1.2
BF:BW (g/kg)	4.28 ± 0.09	4.14 ± 0.14	4.1 ± 0.1	3.9 ± 0.3
SDF weight (g)	2.06 ± 0.17	1.97 ± 0.09	2.5 ± 0.3	3.5 ± 0.6
SDF:BW (g/kg)	0.67 ± 0.03	0.67 ± 0.03	0.7 ± 0.05	0.9 ± 0.2
Muscle biochemical composition				
BF water content (%)	82.1 ± 0.2	80.5 ± 0.2^c^	79.1 ± 0.3	80.8 ± 0.2^a^
BF protein content				
(mg/g wet weight)	44.0 ± 1.2	47.5 ± 2.6	52.5 ± 2.9	40.8 ± 4.3^a^
(mg/mg dry weight)	0.25 ± 0.01	0.24 ± 0.02	0.25 ± 0.01	0.21 ± 0.02
SDF water content (%)	80.9 ± 0.3	79.8 ± 0.5	79.5 ± 0.2	81.2 ± 0.2^a^
SDF protein content				
(mg/g wet weight)	51.1 ± 2.1	50.9 ± 2.3	30.4 ± 0.8	28.9 ± 0.5
(mg/mg dry weight)	0.27 ± 0.01	0.25 ± 0.01	0.15 ± 0.003	0.15 ± 0.002

Significantly different from the value in the control fetuses at the same gestational age where ^a^*P* < 0.05; ^b^*P* = 0.057 (*t*-test or non-parametric Mann–Whitney test as appropriate); ^c^*P* < 0.01.

Neither cortisol infusion nor AX had a significant effect on fetal morphometric measurements or muscle weights compared with their respective controls (Table 2). Water content was significantly lower in the BF of cortisol-infused than saline-infused fetuses at 129 dGA and significantly higher in both muscles of AX compared to sham-operated fetuses at 144 dGA (Table 2). Cortisol infusion had no effect on protein content of either muscle, whereas AX reduced the protein content of the BF alone when expressed per gram wet weight but not per gram dry weight (Table 2). Cortisol infusion has no effect on the fetal blood gas status or concentrations of glucose and lactate during the infusion period before tissue collection (data not shown).

### Muscle mitochondrial content

In control fetuses, CS activity increased towards term with a further increase after birth in both muscles (Fig. 1B and C), consistent with previous findings in the BF (Davies *et al.* 2020). Cortisol infusion had no effect on CS activity in the BF but significantly increased activity in the SDF relative to saline-infused control values (Fig. 1B and C). In contrast, CS activity was significantly less in AX than sham-operated fetuses near term in the BF but not in the SDF when comparing group means (Fig. 1B and C). However, a paired comparison between the AX fetus and its sham-operated twin showed CS activity was significantly less in the AX twin than its sibling for both the BF (−0.090 ± 0.006 μmol/min/mg protein, *n* = 4, *P* < 0.01) and SDF (−0.040 ± 0.008 μmol/min/mg protein, *n* = 4, *P* < 0.05, *t*-test for significance of single mean, both muscles).

When data from all the groups were combined irrespective of age or treatment, there were significant positive correlations between CS activity and the concentrations of both cortisol and T_3_ in each muscle (Table 3). As the cortisol and T_3_ concentrations were also correlated (*r* = 0.720, *n* = 33, *P* < 0.001), partial correlation analyses were used to determine the relative importance of the two hormones when the confounding effect of the other was taken into account. This showed that both hormones have significant influences on CS activity with plasma T_3_ as the more statistically significant factor in both muscles (Table 3).

**Table 3:** Correlation and partial correlation analyses between hormone concentrations and citrate synthase activity and mitochondrial oxidative phosphorylation (OXPHOS) rates of the fetal *biceps femoris* (BF) and *superficial digital flexor* (SDF) muscles. For each muscle, data were combined from the cortisol infused and adrenalectomised and their respective control groups of fetuses.

Muscle	Hormone	Citrate synthase	Py-linked OXPHOS	PC-linked OXPHOS	Maximal OXPHOS
Correlations					
BF	Log_10_ cortisol	*r*** = 0.735** *P* < 0.01 *n* = 33	*r*** = 0.482** *P* < 0.05 *n* = 22	*r* = −0.050 *P* > 0.05 *n* = 19	*R* = 0.263 *P* > 0.05 *n* = 22
	Log_10_ T_3_	*r*** = 0.803** *P* < 0.001 *n* = 33	*r*** = 0.460** *P* < 0.05 *n* = 22	*r* = 0.272 *P* > 0.05 *n* = 19	*R* = 0.304 *P* > 0.05 *n* = 22
SDF	Log_10_ cortisol	*r*** = 0.701** *P* < 0.001 *n* = 32	*r* = 0.203 *P* > 0.05 *n* = 21	*r*** = 0.507** *P* < 0.05 *n* = 19	*r*** = 0.421** *P* < 0.05 *n* = 21
	Log_10_ T_3_	*r*** = 0.801** *P* < 0.001 *n* = 32	*r*** = 0.428** *P* < 0.05 *n* = 21	*r*** = 0.446** *P* = 0.050 *n* = 19	*r*** = 0.566** *P* < 0.01 *n* = 21
Partial correlations					
BF	Log_10_ cortisol	*r*** = 0.387** *P* < 0.05	*r* = 0.350 *P* > 0.05	Not required	Not required
	Log_10_ T_3_	*r*** = 0.582** *P* < 0.01 *n* = 33	*r* = 0.310 *P* > 0.05 *n* = 21	Not required	Not required
SDF	Log_10_ cortisol	*r*** = 0.424** *P* < 0.05	Not required	*r* = 0.390 *P* > 0.05	*r* = 0.215 *P* > 0.05
	Log_10_ T_3_	*r*** = 0.506** *P* < 0.01 *n* = 32	Not required	*r* = 0.279 *P* > 0.05 *n* = 19	*r*** = 0.478** *P* < 0.01 *n* = 21

Significant correlations and partial correlations are shown in bold (*P* ≤ 0.05).

### Muscle mitochondrial biogenesis and membrane dynamics

**Figure 2:**
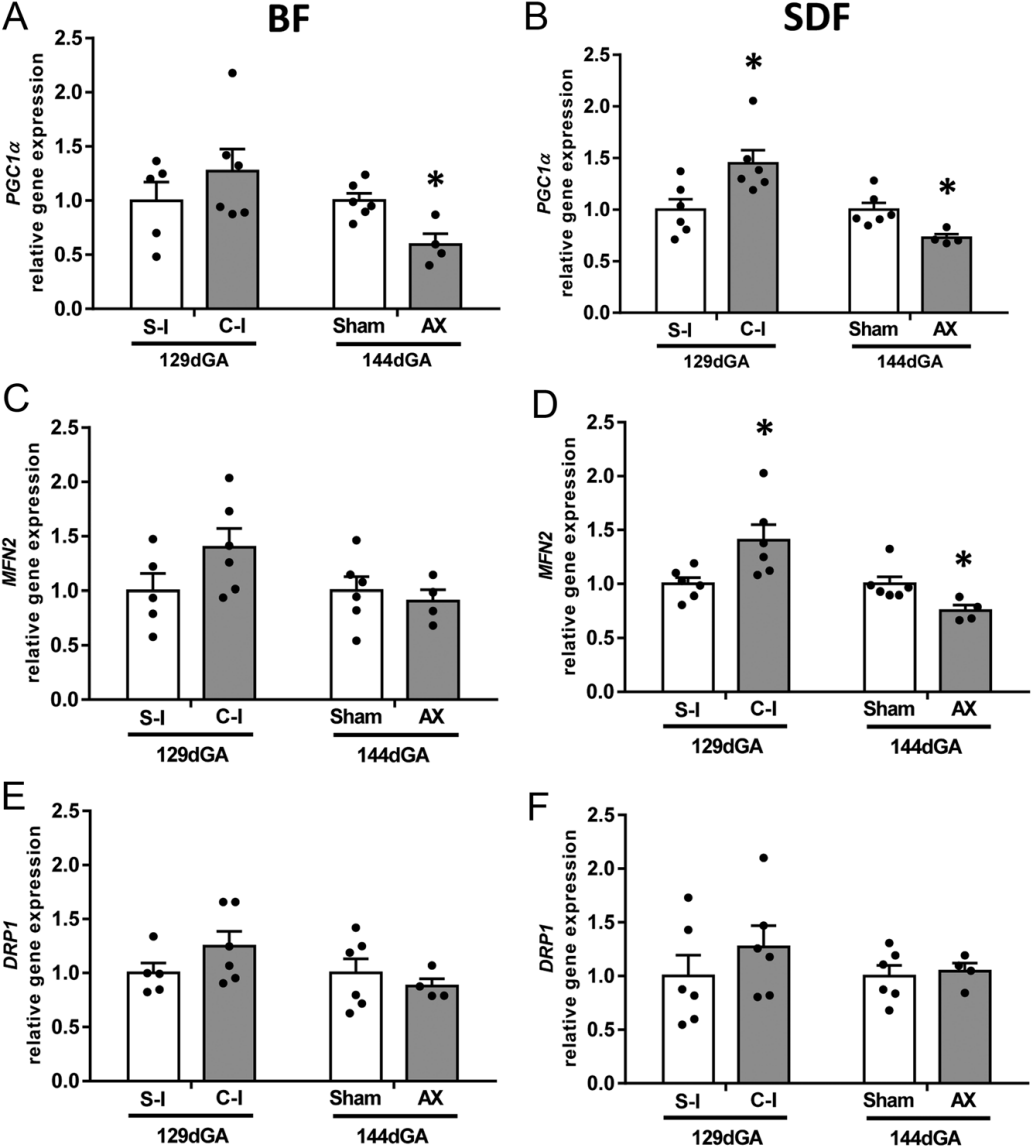
Mean (±s.e.m.) relative gene expression of *PGC1α* (panels A and B), *MFN2* (panels C and D) and *DRP1* (panels E andF) in the *biceps femoris* (BF, panels A, C and E) and *superficial digital flexor* (*SDF*, panels B, D and F) muscles of fetal sheep either at 129 days of gestational age (dGA) after 5 days of infusion of saline (S-I, *n* = 5 BF, *n* = 6 SDF) or cortisol (C-I, *n* = 6, both muscles) or at 144 dGA after adrenalectomy (AX, *n* = 4, both muscles) or sham operation (Sham, *n* = 6, both muscles) at 114–119 dGA. An asterisk indicates a significant difference from the respective control group (*P < 0.05, *t*-test or Mann–Whitney Rank sum test).

Consistent with the changes in mitochondrial density, manipulating fetal cortisol concentrations had muscle-specific effects on gene expression of *PGC1α* and *MFN2*. Expression of *PGC1α* was significantly higher in the SDF of cortisol – than saline-infused fetuses, but not in the BF, and was reduced significantly by AX in both muscles near term (Fig. 2A and B). Expression of *MFN2* in the BF was unaffected by varying cortisol concentrations (Fig. 2C). In contrast in the SDF, *MFN2* expression was upregulated by cortisol infusion and down-regulated by AX relative to their respective controls (Fig. 2D). In both muscles, varying cortisol concentrations had no significant effect on *DRP1* expression (Fig. 2E and F).

### Muscle oxygen consumption

**Figure 3:**
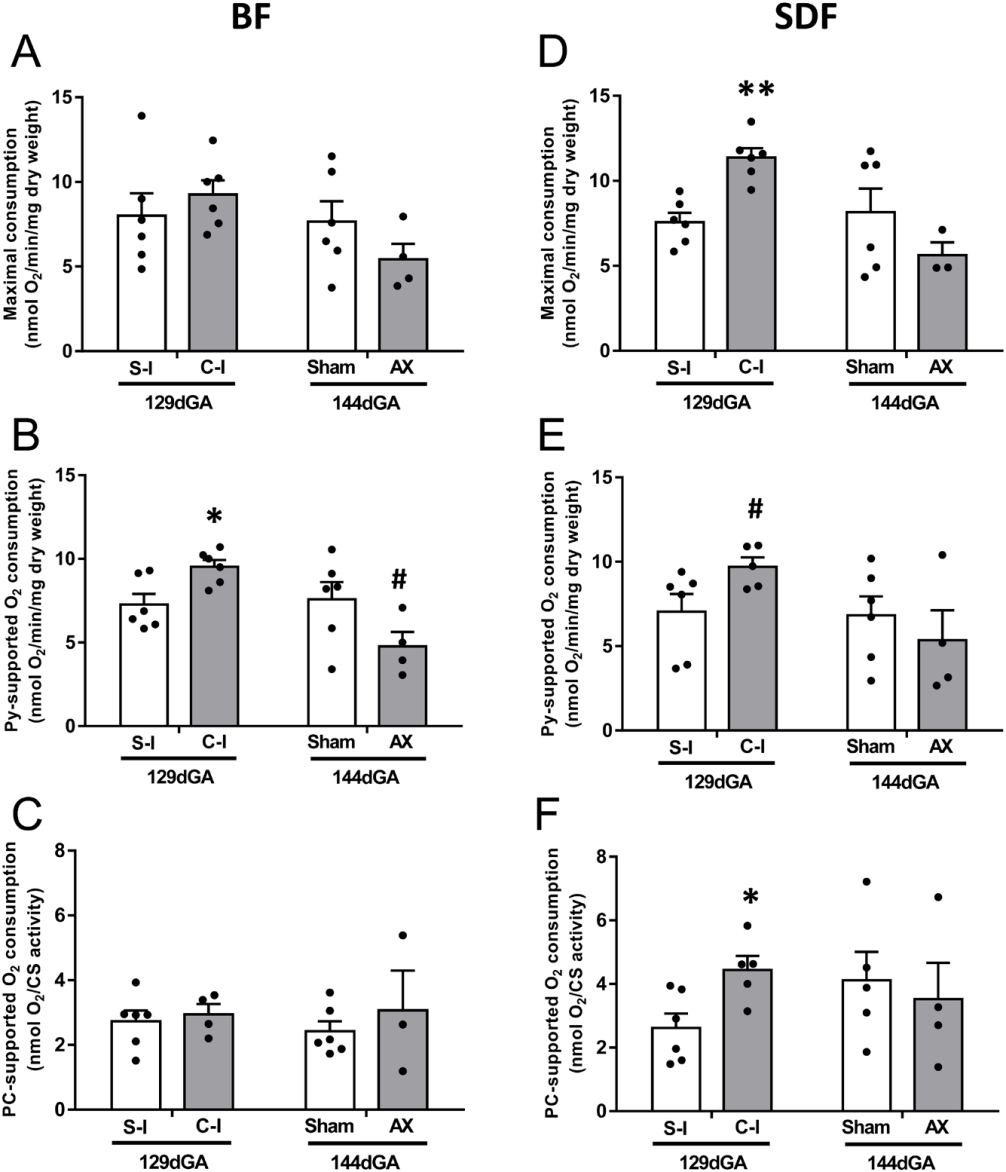
Mean (±s.e.m.) maximal (panels A and D), pyruvate supported (Py, panels B and E) and palmitoyl-carnitine supported (PC, panels C and F) rates of oxygen consumption by the *biceps femoris* (BF, panels A, B and C) and *superficial digital flexor* (SDF, panels D, E and F) muscles of fetal sheep either at 129 days of gestational age (dGA) after 5 days of infusion of saline (S-I, *n* = 6, both muscles) or cortisol (C-I, *n* = 4–6 BF, *n* = 5–6 SDF) or at 144 dGA after adrenalectomy (AX, *n* = 3–4, both muscles) or sham operation (Sham, *n* = 6 BF, *n* = 5–6 SDF) at 114–119 dGA. An asterisk indicates a significant difference from the respective control group (*P < 0.05, **P < 0.01, *t*-test or Mann–Whitney Rank sum test). A hash tag indicates a trend towards a significant difference from the respective control group (^#^*P* < 0.10, *t*-test or Mann–Whitney Rank sum test).

The ADP-coupled rates of O_2_ consumption by the two muscles are shown in Fig. 3 for the three different respiratory protocols. In the BF, cortisol infusion had no effect on maximal OXPHOS or PC-supported oxidative capacity but significantly increased Py-supported O_2_ consumption relative to saline-infused values (Fig. 3A, B and C). In contrast, in the SDF, cortisol infusion significantly increased maximal OXPHOS and PC-supported oxidative capacity together with a tendency for higher rates of Py-supported respiration compared to saline-infused values (*P* = 0.064, Fig. 3D, E and F). In both muscles, AX had no significant effect on respiratory rates using any of the substrates, although there was a tendency for lower BF rates of Py-supported respiration after AX (*P* = 0.093, Fig. 3B).

When the respiratory data were combined for all fetuses irrespective of treatment or gestational age for each substrate separately, there were significant positive correlations between the BF rate of Py-linked respiration and the concentrations of both cortisol and T_3_, although partial correlation showed no significant correlations with either hormone alone (Table 3). There were no significant correlations between the BF respiratory rates with the other substrates and either hormone concentration (Table 3). In the SDF, cortisol concentrations were positively correlated with PC-linked respiration and maximal OXPHOS but not Py-linked respiration while T_3_ levels were positively correlated to all three respiratory rates (Table 3). Partial correlation of the SDF data showed no effect of either cortisol or T_3_ alone on PC-linked respiration but a statistically dominant effect of T_3_ on maximal OXPHOS (Table 3).

In the BF, leak state respiration, a measure of O_2_ consumption for processes other than ATP production, was unaffected by manipulating fetal cortisol concentrations, irrespective of substrate (data not shown). In the SDF, leak state respiration with PC was significantly higher in cortisol – (1.30 ± 0.21 nmolO_2_/min/mg dry weight, *n* = 5) than saline-infused fetuses (0.69 ± 0.17 nmolO_2_/min/mg dry weight, *n* = 6, *P* < 0.05) but not with the other substrates (data not shown). Adrenalectomy had no significant effect on the SDF leak state respiration using any of the substrates (data not shown). There were no significant correlations between any of leak state respiratory rates and the concentrations of either hormone (*P* > 0.05, all cases).

### ETS and other mitochondrial OXPHOS regulatory proteins

**Figure 4:**
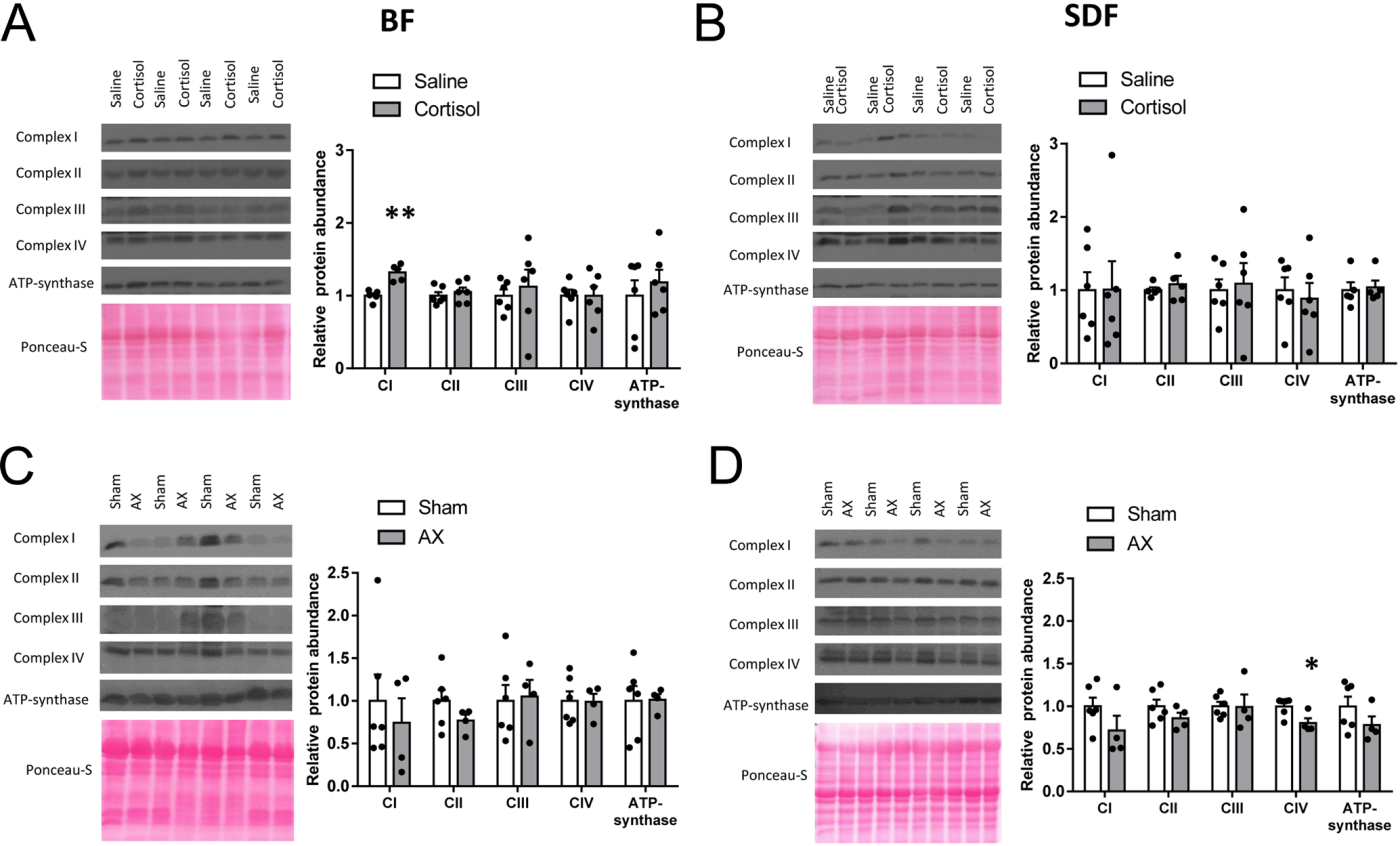
Mean (±s.e.m.) relative protein abundance of the electron transfer system complexes (CI-IV) and ATP synthase (CV) in the *biceps femoris* (*BF*, panels A and C) and *superficial digital flexor* (*SDF*, panels B and D) muscles in fetal sheep either at 129 days of gestational age (dGA) after 5 days of infusion of saline (white columns, *n* = 5 BF, *n* = 6 SDF) or cortisol (grey columns, *n* = 6, both muscles) in panels A and B or at 144 dGA after adrenalectomy (AX, grey columns, *n* = 4, both muscles) or sham operation (white columns, *n* = 6, both muscles) at 114–119 dGA in panels C and D. An asterisk indicates a significant difference from the respective control group (*P < 0.05, **P < 0.01, *t*-test or Mann–Whitney Rank sum test). A full colour version of this figure is available at https://doi.org/10.1530/JOE-21-0171.

**Figure 5:**
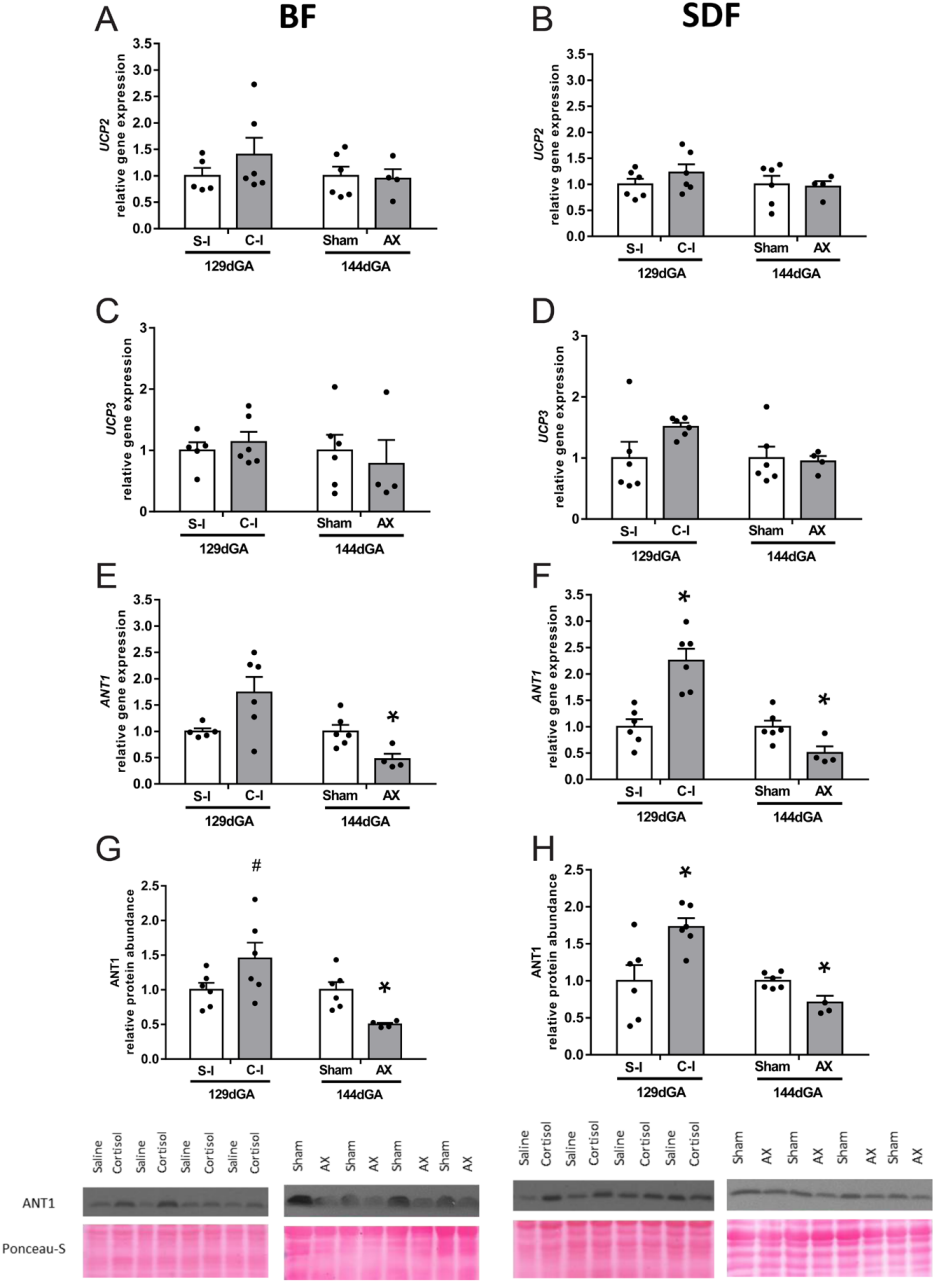
Mean (±s.e.m.) relative gene expression of *UCP2* (panels A and B), *UCP3* (panels C and D), and *ANT1* (panels E and F) and of ANT1 protein abundance (panels G and H) in the *biceps femoris* (*BF*, panels A, C, E and G) and *superficial digital flexor* (*SDF*, panels B, D, F and H) muscles of fetal sheep either at 129 days of gestational age (dGA) after 5 days of infusion of saline (S-I, *n* = 5 *BF*, *n* = 6 *SDF*) or cortisol (C-I, *n* = 6, both muscles) or at 144 dGA after adrenalectomy (AX, *n* = 4, both muscles) or sham operation (Sham, *n* = 6, both muscles) at 114–119 dGA. An asterisk indicates a significant difference from the respective control group (*P < 0.05, **P < 0.01, *t*-test or Mann–Whitney Rank sum test). A hash tag indicates a trend towards a significant different from the respective control group (^#^*P* < 0.10, *t*-test or Mann–Whitney Rank sum test). A full colour version of this figure is available at https://doi.org/10.1530/JOE-21-0171.

In the BF, cortisol infusion significantly increased protein abundance of ETS complex I but had no effect on any of the other complexes or ATP synthase (Fig. 4A). Complexes I-IV and ATP synthase were also unaffected by cortisol infusion in the SDF (Fig. 4B). In contrast, AX had no significant effect on protein abundance of complexes I-IV or ATP synthase in the BF but reduced complex IV abundance alone in the SDF relative to sham-operated values (Fig. 4C and D). Gene expression for the uncoupling proteins, UCP2 and UCP3, was unaffected by treatment in both muscles (Fig. 5A, B, C and D). In the SDF, cortisol infusion significantly increased both gene expression and protein abundance of ANT1 whereas, in the BF, it had no significant effect on either ANT1 measure, although there was a tendency for higher protein abundance relative to saline-infused values (*P* = 0.095, respectively, Fig. 5E, F, G and H). Adrenalectomy reduced gene and protein ANT1 levels significantly in both muscles (Fig. 5E, F, G and H).

### Muscle expression of myosin heavy chain (*MHC*) isoforms

**Figure 6:**
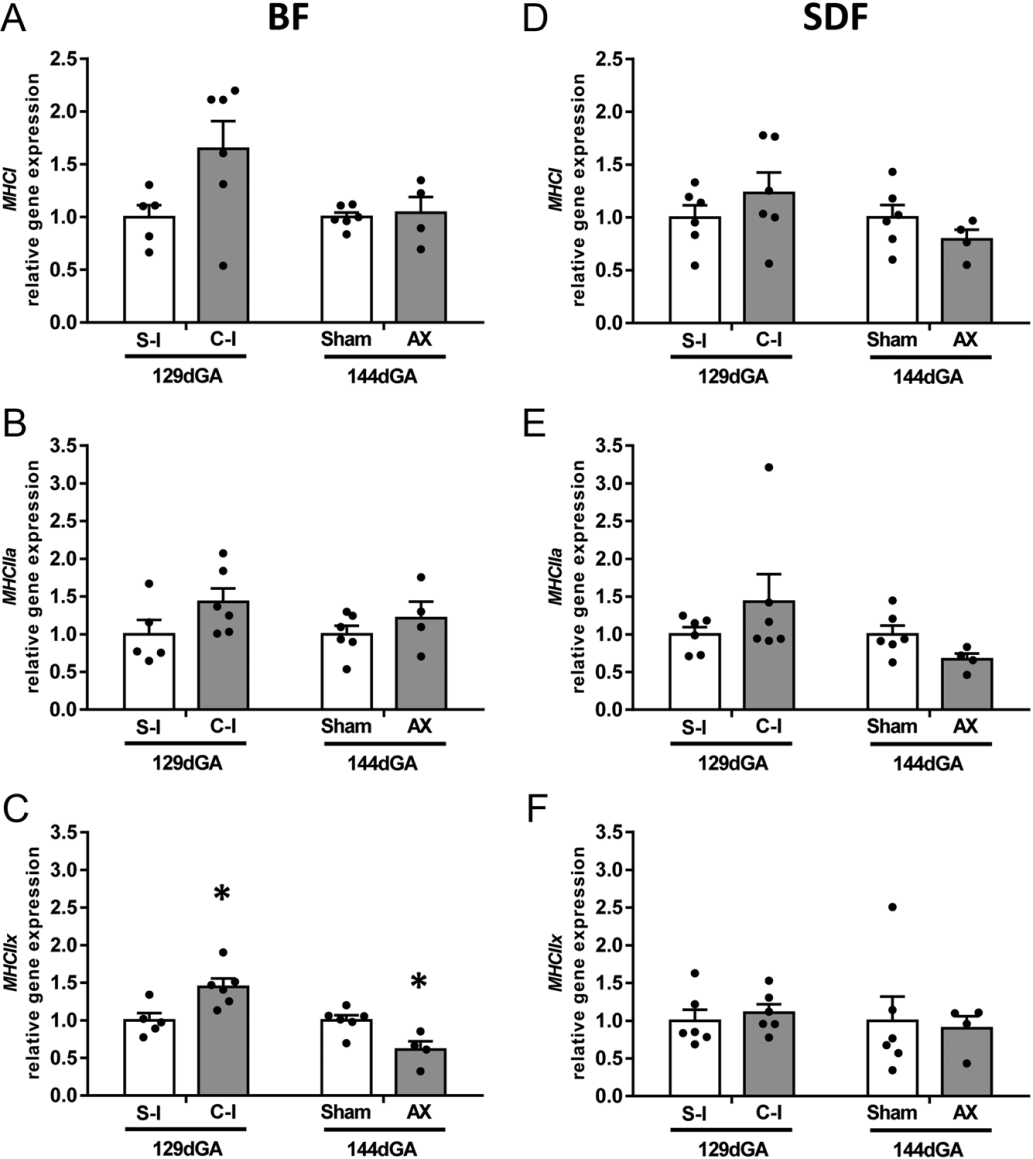
Mean (±s.e.m.) relative gene expression of *MHCI* (panels A and D), *MHCIIa* (panels D and E), and *MCHIIx* (panels C and F) in the *biceps femoris* (BF, panels A, B, C) and (C) *superficial digital flexor* (SDF, panels D, E, F) muscles of fetal sheep either at 129 days of gestational age ( dGA) after 5 days of infusion of saline (S-I, *n* = 5 *BF*, *n* = 6 *SDF*) or cortisol (C-I, *n* = 6, both muscles) or at 144 dGA after adrenalectomy (AX, *n* = 4, both muscles) or sham operation (Sham, *n* = 6, both muscles) at 114–119 dGA. An asterisk indicates a significant difference from the respective control group (*P < 0.05, *t*-test or Mann–Whitney Rank sum test).

The effects of manipulating the fetal cortisol concentration on fibre composition of the two muscles was assessed by quantifying *MHC* isoform expression for the type 1 slow-twitch, oxidative fibres with abundant mitochondria (*MHCI*) and the type II fast-twitch fibres that have fewer mitochondria and are either oxidative/glycolytic, *MHCIIa*, or predominantly glycolytic, *MHCIIx* (Yates *et al.* 2016). In both muscles, cortisol infusion had no significant effect on expression of the *MHCI* or *MHCIIa* isoforms (Fig. 6A, B, D and E). In contrast, *MHCIIx* expression in the BF was increased by cortisol infusion and decreased by AX relative to their respective controls (Fig. 6C). No change in *MHCIIx* expression was seen in the SDF with either treatment (Fig. 6F).

## Discussion

The results show that variations in fetal cortisol concentrations within the physiological range affect mitochondrial OXPHOS capacity in ovine skeletal muscles near term. The effects were muscle-specific and were associated with changes in mitochondrial content, biogenesis markers and abundance of specific ETS complexes and ANT1. They were accompanied by substrate-specific alterations in respiratory function. In addition, there were muscle-specific changes in *MHC* isoform expression in response to altering fetal cortisol concentrations. The cortisol-dependent changes in mitochondrial function are summarised in Table 4 for the two muscles. Collectively, they indicate that the normal prepartum rise in fetal cortisol concentrations has a key role in maturing mitochondrial capacity in preparation for the increased energy demands of skeletal muscle postnatally.

**Table 4:** Summary of the changes induced in the *biceps femoris* and *superficial digital flexor* muscles either by pre-term cortisol infusion for 5 days to mimic the normal prepartum increase in cortisol concentration or by adrenalectomy (AX) to prevent this increment near term relative to their respective control groups.

	Biceps femoris		Superficial digital flexor	
	Cortisol-infused	AX	Cortisol-infused	AX
Mitochondrial density and biogenesis				
Citrate synthase	No Δ	↓	↑	↓^a^
*PGC1a*	No Δ	↓	↑	↓
*MFN2*	No Δ	No Δ	↑	↓
*DRP1*	No Δ	No Δ	No Δ	No Δ
Mitochondrial ADP-coupled respiration				
Pyruvate (Py)	↑	Tendency ↓	Tendency ↑	No Δ
Palmitoyl-carnitine (PC)	No Δ	No Δ	↑	No Δ
Total	No Δ	No Δ	↑	No Δ
Mitochondrial OXPHOS efficiency				
ETS complexes & ATP synthase	↑ CI	No Δ	No Δ	↓ CIV
*UCP2* &* UCP3*	No Δ	No Δ	No Δ	No Δ
*ANT1* gene	No Δ	↓	↑	↓
ANT1 protein	Tendency ↑	↓	↑	↓
Muscle fibre composition				
*MHCI*	No Δ	No Δ	No Δ	No Δ
*MHCIIa*	No Δ	No Δ	No Δ	No Δ
*MHCIIx*	↑	↓	No Δ	No Δ

↓Significant decrease (*P* < 0.05) or tendency for decrease (*P* < 0.10); ^a^Significant decrease by paired t-test to its sham-operated twin (*P* < 0.05); ↑Significant increase (*P* < 0.05) or tendency for increase (*P* < 0.10).

CI, ETS complex I; CIV, ETS complex IV; No Δ, no significant change.

In the current study, mitochondrial content was reduced in both muscles when the normal prepartum cortisol surge was prevented by fetal AX. In rats, suppressing fetal corticosterone concentrations close to term by maternal AX and metopirone treatment reduces mitochondrial content of the fetal kidney but not the liver or heart (Prieur *et al.* 1998). Short-term maternal administration of a potent synthetic glucocorticoid, dexamethasone, near term, restored the normal renal mitochondrial density in these glucocorticoid-deficient rat pups and also increased the volume density of mitochondria in type II pneumocytes of normal fetal rabbits (Snyder *et al.* 1992, Prieur *et al.* 1998). In the current study, raising cortisol level to prepartum values by cortisol infusion before the normal surge increased mitochondrial content, specifically in the SDF. In a recent study, longer-term treatment of pregnant ewes with cortisol for the last 25 days of pregnancy reduced mitochondrial DNA content of the fetal BF and heart at term (Joseph *et al.* 2020). Similarly, maternal corticosterone treatment of rats at mid-pregnancy decreased placental mitochondrial density (Bartho *et al.* 2019). In the current study, muscle mitochondrial content increased between 104 and 129 dGA in the absence of any cortisol increment. This coincides with a major period of muscle fibre differentiation and suggests that factors other than circulating cortisol, such as growth factors and receptor abundances, may be involved in mitochondrial development earlier in gestation (Florini *et al.* 1991, Walker & Luff 1995, Bloise *et al.* 2018). Collectively, these findings suggest that glucocorticoids are required for normal mitochondrial biogenesis near term in specific fetal tissues but that, earlier in gestation, their actions may depend not only on the tissue and its stage of development but also on the duration, timing, route and type of glucocorticoid exposure.

The changes in muscle mitochondrial density seen in response to varying fetal cortisol levels in the current study tracked closely with the expression of the key regulator of mitochondrial biogenesis, *PGC1α* (Table 4). Alterations in *PGC1α* expression were more pronounced in the SDF than BF and were accompanied by parallel changes in SDF expression of *MFN2*, a gene essential for normal membrane dynamics and OXPHOS function that is regulated by *PGC1α* (Liang & Ward 2006). Previous studies on rodents have shown that *PGC1α* expression is glucocorticoid sensitive and increases towards term in fetal heart and adipose tissue (Rog-Zielinska *et al.* 2015, Chen *et al.* 2020). Deletion of *PGC1α* expression in fetal mice also impairs mitochondrial OXPHOS function and the metabolic response to glucocorticoids in developing cardiomyocytes (Rog-Zielinska *et al.* 2015). Conversely, over-expression of *PGC1α* promotes mitochondrial biogenesis and O_2_ consumption in neonatal cardiomyocytes *in vitro* (Lehman *et al.* 2000). However, no prepartum upregulation of *PGC1α* expression was seen in fetal ovine BF, despite a concomitant increase in mitochondrial density towards term (Davies *et al.* 2020).

Previous rodent studies have shown increases in mitochondrial respiration and/or expression of complex IV and ATP synthase in heart, liver and brain of fetal and neonatal pups in response to dexamethasone treatment (Prieur *et al.* 1998, Lehman *et al.* 2000, Nakai *et al.* 2002, Rog-Zielinska *et al.* 2015). In the present study, raising cortisol levels within the physiological range increased mitochondrial OXPHOS capacity in both fetal skeletal muscles but in a substrate-specific manner. In the BF, cortisol stimulated respiration with pyruvate by 30% but not with the other substrates. This occurred without any significant change in mitochondrial content but was accompanied by a similar percentage increase in complex I abundance, consistent with pyruvate being an electron donor to this complex via NADH (Kuznetsov *et al.* 2008). There was, however, no accompanying increase in maximal OXPHOS capacity, supported by saturating concentrations of substrates for complex I and complex II, which may be due to limitations at the Q-junction for electron entry to complex III, which did not increase in abundance. Cortisol-induced upregulation of Py-linked respiration in the BF was also accompanied by greater *MCHIIx* expression consistent with the increased BF abundance of *MCHIIx* glycolytic fibres seen previously towards term (Davies *et al.* 2020). Collectively, the current findings in the BF may suggest that the mitochondrial content of its oxidative fibres increases in response to cortisol infusion. In contrast, in the SDF, cortisol infusion resulted in significant rises in PC-linked and maximal OXPHOS capacity together with a tendency for higher rates of Py-supported respiration (Table 4). These respiratory changes occurred without alteration in *MHC* expression but concomitantly with increased mitochondrial biogenesis and content. However, preventing the prepartum fetal cortisol surge by AX had no significant effect on mitochondrial respiration in either muscle irrespective of substrate, despite decreased expression of *MHCIIX* in the BF and lower mitochondrial density and *PGC1α* expression in both muscles. Thus, cortisol appears to act on mitochondrial OXPHOS *in utero* through different muscle-specific mechanisms, which may also depend on gestational age.

The discrepancy between the effects of cortisol on respiratory rates at 129 and 144 dGA may reflect, in part, differences in the duration of cortisol exposure between the single infused and twin sham-operated fetuses as activation of the fetal hypothalamic–pituitary–adrenal axis, and the rise in fetal cortisol concentrations occurs more rapidly and closer to term in twin than single sheep fetuses (Edwards & McMillen 2002). Since cortisol activates the deiodinases converting T_4_ to T_3_ (Forhead *et al.* 2006), the current findings that fetal T_3_ concentrations were increased by 5 days of cortisol infusion but did not differ significantly between sham-operated and AX fetuses later in gestation would be consistent with a shorter period of cortisol exposure in the sham-operated twin fetuses. Thyroid hormones are known to affect mitochondrial function in adult tissues and their fetal deficiency has recently been shown to impair mitochondrial OXPHOS capacity of the fetal ovine BF and brain (Lombardi *et al.* 2015, Bloise *et al.* 2018, Davies *et al.* 2020, 2021). Indeed, the current findings suggest that both cortisol and T_3_ are important factors in regulating mitochondrial content and OXPHOS capacity of skeletal muscle during late gestation. The prepartum maturational effects of cortisol on mitochondrial function in skeletal muscle may, therefore, be mediated, in part, by T_3_ as occurs with other metabolic processes which are essential for neonatal survival (Forhead & Fowden 2014).

The current findings in AX fetuses indicate that the prepartum cortisol increment increases mitochondrial content in both muscles. However, earlier in gestation when muscle fibres were still differentiating, the effects of cortisol are more complex and appear to be muscle and possibly fibre-type specific. In the SDF, cortisol infusion increased mitochondrial biogenesis, content and maximal OXPHOS, but with no apparent increase in ETS complex abundance. In the BF, cortisol infusion had no effect on mitochondrial content or maximal OXPHOS, but specifically increased complex I capacity and altered the relative contribution of the different muscle fibres to the mitochondrial pool. In both muscles, there were no changes in ATP synthase or UCPs with experimental manipulation of the fetal cortisol concentration that would explain the changes in OXPHOS functional capacity, although UCP expression may not reflect the activity. This contrasts with the known effects of cortisol in upregulating UCP abundance in fetal ovine adipose tissue near term (Mostyn *et al.* 2004, Gnanalingham *et al.* 2008). In general, ANT1 levels were increased by cortisol infusion and reduced by AX in both muscles in the current study. As well as functioning as a mitochondrial ADP-ATP exchanger, ANT1 can induce mild mitochondrial uncoupling in adult tissues, particularly in response to fatty acids (Kimura & Rasmussen 1977, Brand *et al.* 2005, Sparks *et al.* 2016). This is consistent with the current finding of greater ANT1 abundance concurrently with increased SDF rates of both PC-linked leak and OXPHOS respiration in cortisol-infused fetuses. In adult rat liver, dexamethasone has been shown to increase ANT1 content and simultaneously enhance both mitochondrial uncoupling and OXPHOS capacity (Arvier *et al.* 2007). The prepartum rise in cortisol may, therefore, act to stimulate mitochondrial biogenesis and, thus, the capacity for neonatal ATP production while minimising the potential for oxidative stress, in part through dissipating the proton gradient. Other factors may then activate the increase in mitochondrial OXPHOS after birth when the ATP demand rises with the new metabolic activities (Fowden & Forhead 2015).

In summary, the current findings show that cortisol is an important regulator of mitochondrial OXPHOS capacity in the ovine skeletal muscle during late gestation. Its effects were muscle-specific and involved changes in mitochondrial biogenesis and respiratory function. Indeed, these prenatal cortisol-induced adaptations may explain, in part, the adult mitochondrial dysfunction observed after adverse conditions during pregnancy that raises fetal glucocorticoid concentrations (Reynolds 2013, Khamoui *et al.* 2018, Chen *et al.* 2020, Gyllenhammer *et al.* 2020). While glucocorticoids are known to affect adult mitochondrial function through both the nuclear and mitochondrial genomes (Lapp *et al.* 2019), further studies are needed to determine the specific molecular mechanisms by which cortisol induces mitochondrial maturation in skeletal muscle fibres. Greater knowledge of these developmental processes will be beneficial for the metabolic health of infants under- or over-exposed to glucocorticoids prenatally due to stress, prematurity or maternal treatment with synthetic glucocorticoids for threatened pre-term delivery or other clinical conditions.

## Declaration of interest

The authors declare that there is no conflict of interest that could be perceived as prejudicing the impartiality of the research reported.

## Funding

The work was supported by a grant to A L F and A J M by the Biotechnology and Biological Sciences Research Council http://dx.doi.org/10.13039/501100000268 (BB/P019048/1).

## Author contribution statement

The study was designed by K L D, O R V, A J M and A L F. The *in vivo* experimental work on the animals was carried out by K L D, E J C, D J S, A J F and A L F. The *in vitro* tissue analyses were carried out by K L D, D J S, E J C and A J M. The manuscript was written by K L D and A L F. All the other authors commented on the text.
